# The good, the bad, and the ugly of metals as antimicrobials

**DOI:** 10.1007/s10534-023-00565-y

**Published:** 2023-12-19

**Authors:** Raymond J. Turner

**Affiliations:** Department of Biological Sciences, University of Calgary, 2500 University Dr. NW, Calgary, AB Canada

**Keywords:** AMR, Antibiotics, Resistance, One Health, Metals, Antimicrobial metals, Metallobiotics, Metalloantibiotic, Metal complexes, Nanomaterial, Toxic metals, Heavy metals

## Abstract

We are now moving into the antimicrobial resistance (AMR) era where more antibiotic resistant bacteria are now the majority, a problem brought on by both misuse and over use of antibiotics. Unfortunately, the antibiotic development pipeline dwindled away over the past decades as they are not very profitable compounds for companies to develop. Regardless researchers over the past decade have made strides to explore alternative options and out of this we see revisiting historical infection control agents such as toxic metals. From this we now see a field of research exploring the efficacy of metal ions and metal complexes as antimicrobials. Such antimicrobials are delivered in a variety of forms from metal salts, alloys, metal complexes, organometallic compounds, and metal based nanomaterials and gives us the broad term metalloantimicrobials. We now see many effective formulations applied for various applications using metals as antimicrobials that are effective against drug resistant strains. The purpose of the document here is to step aside and begin a conversation on the issues of use of such toxic metal compounds against microbes. This critical opinion mini-review in no way aims to be comprehensive. The goal here is to understand the benefits of metalloantimicrobials, but also to consider strongly the disadvantages of using metals, and what are the potential consequences of misuse and overuse. We need to be conscious of the issues, to see the entire system and affect through a OneHealth vision.

## Introduction

Antibiotics, the 20th-century miracle drugs, have been failing to work for us this century with increased evolved resistance by all pathogens to the existing antimicrobials. This has led towards a prediction of an apocalyptic post-antibiotic life (WHO 2014), reflecting the danger of dying from infection similar to the life before Joseph Lister’s use of antiseptics in surgery in the late 1800s and the discover of antibiotics by Alexander Fleming in 1928. The United Nations predicts that by 2050, someone will die from an antibiotic-resistant superbug every 3 s. Unfortunately, we are already almost there, as a recent study identified that in 2019, 4.95 million deaths worldwide were associated with Antimicrobial Resistance (AMR) bacteria, and 1.27 million were attributed directly to AMR (Hamadani et al. 2022). This is attributed to misuse and overuse of antibiotics which leads us to what is now referred to as the Antimicrobial Resistance (AMR) era. One of the sources of this problem is illustrated in a recent assessment in our local region of Canada (Alberta; population 4.4 million) with nearly 40% of antibiotic prescriptions dispensed to 1.35 million adult patients in Alberta’s community-based settings, over 35 months, were inappropriate leading to the overprescribing of antibiotics such as amoxicillin, azithromycin, and clarithromycin (Leslie et al. 2023).

Of course, this leads to a call for increased antimicrobial stewardship and directed policies around present and future uses, but also a call for alternatives to traditional antibiotics (Dodgostar 2019). Stewardship practices must be present in the discovery pipeline, such as evaluating new antimicrobials for their state of evolved resistance. One must also understand the collateral effects of synergy and antagonism between other compounds, drugs, and metabolites. Antimicrobial practices in healthcare, industry and agriculture need to consider a OneHealth viewpoint (Shakoor et al. 2019, OneCDC 2020) as well as the full system cycle of a compound from synthesis to disposal. We need modified and restricted practices around antimicrobials in industries, their use as preservatives, production of antimicrobials, use in cosmetics and food packaging, use in agriculture and animal husbandry, use of biocides of biofouling control, responsible use of antiseptic cleaners, etc., to just name a few areas.

As alternatives to antibiotics, there is now considerable academic research into various natural and synthetic compounds and other strategies to fight pathogenic bacteria (Plotniece et al. 2023). Examples of novel antimicrobials being explored include: bacterial phage (Schwarz et al 2022), colicins and tailocins (Brown et al 2012), quorum sensing inhibitors (Bhardwaj et al 2013), antimicrobial peptides (Magana et al 2020), cyclic peptides (Lai et al 2022), macrocyclics (Garcia Jimenez et al 2023), repurposing orphan drugs (Boyd et al 2021), searching the microbial dark matter for antibiotic-producing strains (Shukla et al 2023), exploiting bacterial parasitic species such as bespoke predators like *Bdellovibrio*, improving existing antibiotics by co-administering resistance inhibitors or antibiotic resistance breakers (Laws et al 2019), considering physiology modulators and actuators, developing inhibitors for multidrug resistance efflux pumps (Sharma et al 2019), biofilm prevention through material science engineered antimicrobial surfaces and coatings (Francolini and Donelli 2010). Other ideas consider lipid vesicles for targeted delivery and other nanotechnology (Mubeen et al 2021) for delivering vaccines, antibodies, and CRISPR-Cas9 gene editing. And finally revisiting ‘old-school’ approaches such as maggots, natural plant compounds (Winska et al. 2019) and potentially toxic metals. Note, here we will avoid the term 'heavy metals' which is a non-sensical chemical term that we need to remove from our lexicon as per discussions by Duffus (2002) and Pourret et al., (2019, 2021).

Many of the approaches listed above to deal with AMR are either looking at natural antimicrobial processes or revisiting old knowledge. We see alongside natural plant compounds such as poultices, that metal elements have also been used since antiquity. Persians used vessels made of copper or silver to prevent water from fouling. Similar practices were adopted by later civilizations of Phoenicians, Romans, Greeks, and Egyptians (Alexander 2009). Even from the Middle Ages, sailors and American settlers used silver coins in containers of water and milk to prevent dysentery (Borkow and Gabbay 2009). The use of metals in medicine was documented in the Edwin Smith papyrus, the oldest known surgical text dated around 1500 BC. Metals such as tellurium, arsenic, silver, mercury, and copper, were all used for the treatment of wounds and infection control and disease treatment for leprosy, tuberculosis, gonorrhea, syphilis, and anemia (Pereira 1836; Hodges 1889). We see evidence that from the 1700s metals such as copper were used to control fungi on grain seeds and other crop blights (Russell 2005). More recently, we’ve seen metals used as wood preservatives such as mixtures of chromate-arsenic with copper or mercury (Townsend and Sololo-Gabriele 2006).

From this history and examples, we see that the use of metals as antimicrobials is nothing new. They just lost their title to organic chemistry and the discovery of antibiotics and antiseptics. Yet, now, due to the high amount of antimicrobial resistance in all pathogens, we see a renaissance in the exploration of metal-based antimicrobials (MBAs) (Turner 2017; Salazar-Alemán and Turner 2022). In order to get a feeling for the research trends in the field, upon exploring manuscripts in PubMed for the first year of a report on common metals historically used as antimicrobial shows Cu(1943), Ag(1945), Al(1945), Se(1946), Zn(1946), Au(1947), Ni(1950), Ga(1954), Ti(1961). In the case of Bi(1946), we see a peak at 1995 followed by loss of interest to a low in 2003 then an exponential increase. For Hg(1945) and As(1945) we see a slow linear interest compared to the others that instead begin to shoot up exponentially in the early 1990’s and peaked in the past 2–5 years. But these dates are misleading as they reflect the date of understanding that they are acting on specific bacteria causing infection or disease. There are of course much earlier reports of use as of metals as an antimicrobial without an understanding of how it worked, such as the treatment of gunshot wounds with ZnCl_2_ (DeMorgan 1870).

This critical opinion mini review in no way aims to be comprehensive. In the literature we see metal salts, alloys, metal complexes, organometallic compounds, and metal nanomaterials explored as antimicrobials. Several terms have been used to describe them including metal-based antimicrobial (MBA) (traditionally referring to metal ions, metal salt or alloy), metallobiotics (which would be best used to describe metal complexes used as a general biocide) or metalloantibiotic (a metal complex with antibiotic like properties; i.e. a defined single biological target). To encompass all of these, we will use here a broad term of metal-antimicrobial or metalloantimicrobial to capture all uses of metals in many forms to control bacteria and fungi or even protozoans. The goal in this review is to not only understand the benefits of this group of antimicrobials but to also consider any disadvantages of using metals in antimicrobials, and what the potential consequences of misuse and overuse are and could be.

## The good

Bacteria, co-evolved with the changes in the geochemistry of the Earth, and thus have been exposed to a wide variety of metal species at various concentrations for millennia. This led to the incorporation of many metals into the biochemistry of life, giving us what we refer to as our essential metals such as iron, copper, zinc, and others. Regardless, we see many metals and metalloids to be quite antimicrobial (Li et al. 2021b), and even essential metal elements are toxic at higher concentrations disrupting the homeostasis set in an organism (Chandrangsu et al. 2017). Potentially toxic metals that are, or could be used, as antimicrobials to bacteria are effective at concentration ranges from less than one micromolar to tens of millimolar. The general order trend of metalloantimicrobial efficacy (toxicity to bacteria) from 100’s of nanomolar concentrations are tellurium as TeO_3_^2−^: mercury, Hg^2+^; silver, Ag^+^, gold, Au^3+^; to micromolar concentrations copper, Cu^2+^; zinc, Zn^2+^, nickel, Ni^2+^; bismuth, Bi^3+^; to millimolar concentrations cobalt, Co^2+^; aluminum, Al^3+^; gallium, Ga^3+^, Tungsten as WO_4_^2−^; Manganese, Mn^2+^; Selenium, as SeO_3_^2−^ (Nies 1999; Harrison et al 2004; Gugala et al. 2017; Gugala et al. 2019a, b; Pormohammad et al. 2020). Of course, as seen in these and other studies, this order depends on bacterial species and stain, the experimental and incubation conditions (planktonic vs. biofilm), the media constituents (carbon and energy sources) that dictate the organism’s physiology as well as potentially the metal speciation state (bioavailability of the metal ion). A further factor is how the therapeutic window of the host-pathogen physiology will influence the dosage load and subsequent efficacy of the metalloantimicrobial.

Antimicrobial metals can be delivered to bacteria in a variety of ways such as metal alloys, metal salts and ions, metal nanoparticles, metal complexes and organometallic compounds. The simplest version of the metal complexes with a organic constitutes are metallophores, which consist of low molecular mass organic molecules that provide ligands to a given metal atom in order to help bioavailability of metal-ion nutrients to the organism (Kraemer et al. 2015). The best-known natural metallophores for metal ion nutrient uptake are siderophores (Saha et al. 2016) used for iron uptake and chalkophores for copper (Kenney and Rosenzweig 2018). As such, bacteria have specific importers to facilitate the essential metal delivery by the metallophore into cells. These natural metallophores have inspired the possible idea of switching out the iron with a toxic metal such as gallium, such that the siderophore-Ga complex is brought into the cell delivering the toxin; an approach now referred to as the “Trojan Horse Drug”. This has inspired many groups to develop antimicrobials in this fashion including exploring conjugates of the metallophore with established antibiotics or modifying the antibiotic to become a metallophore itself. An example of this is the galbofloxacin which is a designed ciprofloxacin-seferrichrome siderophore (Pandey et al. 2021). Other examples of metallophores for the Trojan horse approach are reviewed by Weng et al. 2023). A modification of this theme is that the metallophore acts as an ionophore, which would be a molecule that increases the permeability of the lipid membrane for a specific ion, and in this case facilitates the movement of the metal ion across the membrane (reviewed in Frei et al. 2023).

The idea of metal complexes as antimicrobials has garnered a lot of attention recently, particularly by the group of Angelo Frei at University of Queensland in Australia and Mark Blaskovich based in Bern, Switzerland where they founded the community for Open Antimicrobial Drug Discovery (CO-ADD). These antimicrobials may include metal coordinated complexes consist of central metal atom(s) ligated by molecules or ligands or as organometallic compounds where metal–carbon covalent bonds to the metals exist. Their CO-ADD has taken the approach of surveying libraries of metal complexes synthesized for other purposes and screening them for antibacterial properties. Promising hits from these libraries are now called metalloantibiotics (Frei 2020; Frei et al. 2020, 2023), although clear antibiotic properties (defined biochemical targets) have not been defined for most hits. Even before this CO-ADD, small organometallic compounds were being revisited as antimicrobials (reviewed by Patra et al 2012) highlighting a variety of metal centres. Further metal complexes were being recognized particularly those with ruthenium (reviewed by Li et al. 2015). Regardless, this approach has added more and unique metals to the metalloantimicrobial compound list including, but not limited to, manganese, molybdonium, rhodium, ruthenium, rhenium, palladium, tungsten, iridium, platinum, lutetium, and osmium.

Further on the idea of metal complexes Wee Han Ang’s group reviewed transition metal scaffolds highlighting catalytic properties, one of which is the localized release nitric oxide or carbon monoxide via specific metal –CO or –NO complexes that the reactive oxide is provide by ligand dissociation (Weng et al. 2023). These are being referred to as ‘triggered warhead release molecules’. This group also overviews complexes that will catalyze bioorthogonal reactions (new to nature chemistry) that can be catalyzed by metal systems. Similarly a recent review from Waters et al. (2023) on similar metal complexes points to a class of catalytic metallodrugs which are chemical scaffolds of reactive metals. These compounds are designed to be catalytic providing degradation of biomolecules in cells and have a redox-active metal center coordinated with an organic directing group. This biomolecular targeting is facilitated by an aptamer (DNA, peptide, lipid) with a high affinity for the target cell and/or biomolecules.

Other metal antimicrobial delivery vehicles have been explored. Polyoxometalates (POMs) are discrete metal-oxide anion clusters with unique physico-chemical properties leading them to be explored as promising metallodrugs.  A review on POM antimicrobial properties (Bijelic et al. 2018) shows interesting promise of these and further expands the metals that can be used as metalloantimicrobials such as vanadium.  Molecular organic frameworks (MOF) consist of an coordination network that has metal atoms are connected by organic bridging ligands. This allows a framework to be produced upon mixing the appropriate metals and ligands. This generates a framework of metal–ligand–metal–ligand etc. MOFs were not initially considered as drug delivery vehicles, but more around being interesting networks that one can insert other molecules into the voids within the matrix (as reviewed by Yusuf et al. 2022). However, recently several groups have begun to explore MOFs as antimicrobials either as a delivery vehicle of the metal or the organic bridging ligand being an antimicrobial or the combination thereof (Sun et al. 2020; Shen et al. 2020; Li et al. 2021a, b).

Another delivery approach for a mixture of antimicrobials is to combine them together either as co-crystals, ionic co-crystals or coordination polymers (reviewed by Braga et al. 2022). Recent explorations have seen metal–organic coordination polymers successfully explored with an antimicrobial metal and an antimicrobial organic combined (Lehkan et al. 2022). The approach of crystal engineering towards antimicrobials (Braga 2023) has shown success in that this mode of metalloantimicrobial can provide additive, synergistic, or containment stability of the organic metal mixture.

A popular  approach for the delivery of metals as antimicrobials is nanomaterial formulations. Nanomaterials or nanoparticles (NP) are materials with at least one dimension less than 100 nm and such sizing gives them unique properties. Over the past decade or more, NPs have become an increasingly popular strategy towards innovative antimicrobial therapies and new materials with enhanced antimicrobial action (Khan et al. 2020). Most of the NP antimicrobial research has been to explore the NP manufacturing or synthesis process. This would include the type of elements in the metal core, the size, shape, and the NP coating or capping. All these parameters influence not only the NP stability but also their expected application’s efficacy, consequently even the surface charge (Abbaszadegan et al. 2015) and organic composition from biogenic (Piacenza et al. 2018) will influence the antimicrobial efficacy. Beyond the metal core and the cap, researchers also explored NPs of other materials such as lipids, carbohydrates or other polymers to be carriers of the antimicrobial (Mercan, 2022; Liew et al. 2022). From this, we see antimicrobial nano formulations being produced that have antibacterial, antiviral, antifungal or antiparasitic properties. A creative example is capturing NPs in a polymer for antimicrobial coatings to prevent biofilms (Balaure and Grumezescu 2020; Mohanta et al. 2023).

A developing approach is to use metal complexes or NPs for photodynamic therapy (Josefsen and Boyle 2008). In this case, specific wavelength irradiation to the metal material leads to the catalytic production of reactive oxygen–nitrogen-sulphur species (RONSs). The catalytic complex may be a metallophore or more elaborately, a targeted metal NP of metal complex that facilitates photodynamic therapy which allows for the targeted release of RONSs at the point of infection. This approach has been successful in cancer therapy and is now being explored for bacterial infection (Cieplik et al. 2018).

But metals can be antimicrobial without all the additional organic chemistry. Solid alloys of some metals demonstrate contact-killing antimicrobial activity. As such, they are considered for use in infection control of high-touch surface environments. An example of this is replacing stainless steel handrails, carts, light fixtures etc. with copper materials (Salgado et al. 2013). The mechanism of the antimicrobial activity is assumed to be from the bacteria’s presence on the surface causing the decomposition of the metal matrix and release of free-metal ions (Vincent et al. 2018; Chang et al. 2021). The bacteria receive a high-concentration dose of the metal ions thus killing or preventing cell propagation which helps keep the surface microbial-free. This process was termed “oligodynamic effect” by Carl Nägeli in 1893.

From this discussion, we see that metals can be used in a wide variety of ways to produce antimicrobial activity. Consequently, there are several highly applicable uses of metalloantimicrobials. These include but are not limited to: medical catheters, wound creams, wound dressings, bandages, wound ointments, infection control sprays, high-touch surfaces in health care facilities and institutions, in wall paint for infection spread control, ear and eye infection drops, medically required wearable devices (insulin pumps and monitors), dental cavity treatment, sunscreens, medical implants, bio composites, key disease transfer high touch surfaces such as door handles on buses and trains, medical personal protective clothing, control for severe dandruff, skin parasite control, biofilm prevention on dental implants, vaccine preservatives, and water treatment. Also not to forget the good uses in other areas such as agriculture pest control, plant pathogen control, cattle hoof rot and other agricultural animal husbandry applications.

In fundamental toxicology studies, one compares the degree of toxicity to the physicochemical parameters of the metal elements. Such parameters are reduction potential, sulphur-metal compound solubility, ionic radius, polarizability, etc. For the metal ion toxicity to several different bacteria, we see linear correlations to such parameters for planktonic and biofilm growth (Workentine et al. 2008; Lemire et al. 2013; Frankel et al. 2016). In addition to toxicology, the interaction of the metal ions with bacteria follows several basic bio-inorganic principles. Such parameters include its speciation which is influenced by temperature, pH, ionic strength, and reduction potential as well as type of solvent. Electron configuration of the element influences the types of ligands it will accept and resulting complexes it can form which is also dictated by the geometry of the coordination and thus the relative affinities of the ligands involved. These parameters in part influence the Irving Williams series that reflect the affinity of different ligands for different metal atoms. From all this, we see that metal toxicity roughly follows hard-soft acid-based theory and generally, we see the metal toxicity to bacteria trends to metals that are softer acids (reviewed in Lemire et al. 2013; Salazar-Alemán and Turner 2022).

**Fig. 1:**
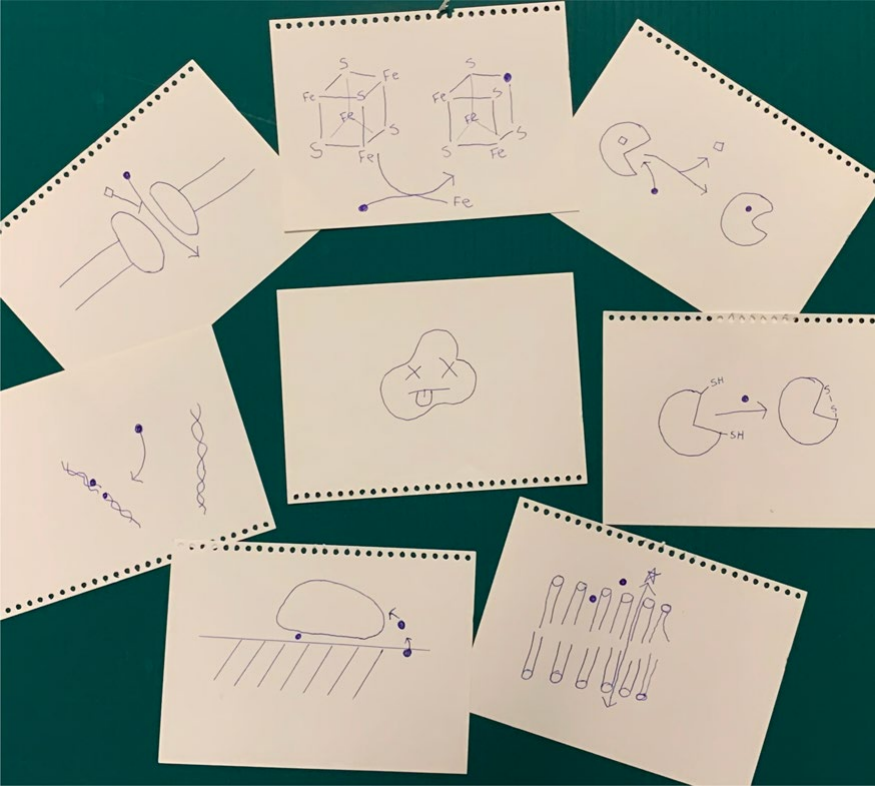
Known mechanisms of toxicity of metals towards bacteria. Here we see cartooned a sick bacteria surrounded by the many possibilities for it to be killed by metals. From the top clockwise: Iron-sulfur center damage, replacement of essential metal in protein or enzyme, oxidation of thiols, membrane damage, contact killing on surface, DNA damage, and out competing essential metal import. Blue ball represents the metal atom, squares the essential metal atom. Of course not to scale

From this bioinorganic chemistry and toxicology as well as biochemical investigations over the past ~ 50 years, there are a number of general molecular mechanisms that have been characterized for how metals act as an antimicrobial. Although similar bio-inorganic chemistry can occur in all organisms, because the cell envelope and the physiology of eukaryotic cells are different than prokaryotic cells, we see very different toxicity levels between these two groups of organisms. The toxicity mechanisms are cartooned in Fig. 1 and text description follows below.

Given one of the most prevalent soft bases in biochemistry is the reduced thiol, we see many of the toxic metals oxidizing the thiol groups and bacteria (Harrison et al. 2009). It is important to note here that as opposed to eukaryotes having a partly oxidized cytoplasm, prokaryotes are fully reduced and thiols are in the RSH form, the oxidation of which generates redox stress to the cell. Another thiol damage mechanism is the destruction of iron-sulphur clusters where the toxic metal competes with the iron in the ligand coordination. The subsequent result is the release of iron ions that then catalyze Fenton reactions leading to reactive oxygen (nitrogen and sulfur) species (ROS) which of course then sets random havoc on biomolecules.

Many metals interfere with the cell wall and envelope in some form. If we consider the lipids of bacteria are primarily negatively charged, one expects electro-static interactions with the lipid headgroups which therefore, affects membrane fluidity, permeability, and function. Another effect on the membrane is interference with nutrient uptake. The metals as ions can compete with essential element uptake in nutrient transporters. The metals as organo-metallic complexes could mimic nutrients and therefore compete for their transport. Some metals such as metalloid oxyanions may steal electrons from the electron transfer chain affecting bioenergetics and potentially also spinning off ROS (Presentato et al. 2019; Kessi et al. 2022).

Once the metal ions are within the cytoplasm, they have access to all the proteins, enzymes and other biomolecules in the cell to bind to. With regards to enzymes, we can consider a toxic metal atom competing with the binding of an essential metal in an enzyme leading to a loss of catalytic activity or a change in function. We can also see metal ions binding to proteins in an allosteric fashion causing folding problems. Finally, the metal ions can interact with DNA causing mutations or strand breaks as well as affecting gene regulation.

Beyond these general mechanisms, there are some specific effects associated with some elements such as arsenic or vanadium competing with phosphate. Where chromate, tellurite and selenite compete with sulphur metabolism. Some metals such as bismuth and chromium bind to peptides such as those in the cell wall. Tungsten specifically competes with molybdenum in molybdopterin containing respiratory enzymes.

Bacteria growing as surface-attached communities are referred to as biofilms. The primary phenotype of biofilms is that they are incredibly antimicrobial-tolerant (reviewed by Davies 2003). This leads to serious challenges in healthcare, in the treatment of infections and diseases associated with biofilms. In 2004, it was discovered that metal ions have the ability to kill bacteria in a biofilm in a time-dependent fashion (Teitzel and Parsek 2003; Harrison et al. 2004). In fact, it was discovered (Harrison et al. 2005) that most metal ions have the ability to kill the problematic persister cells (Lewis 2010) within a biofilm. Thus, solving the antibiotic resistance problem of bacteria growing as a biofilm. In addition to being able to bypass the biofilm antibiotic resistance phenotype, studies often observe that many metal ions are effective against antibiotic resistant and multidrug resistant strains and clinical isolates (Gugala 2017, 2019a; b; Monych et al. 2019). These observations have led to an enhanced interest in metalloantimicrobials in the medical field (further discussed in Gugala and Turner ( 2018).

## The bad

**Fig. 2:**
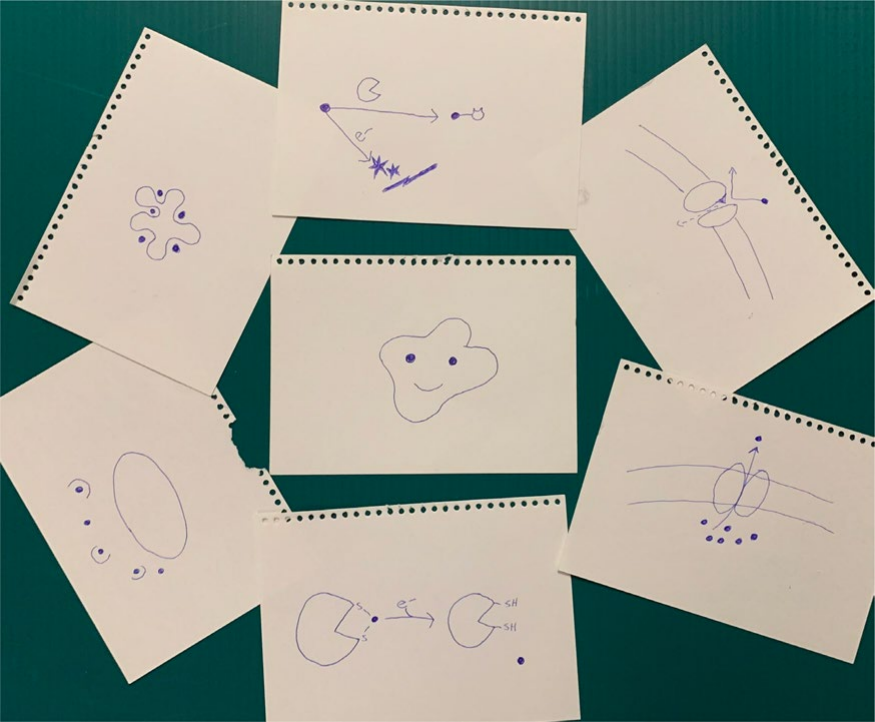
Known mechanisms of resistance/tolerance of metals by bacteria. Here we see cartooned a happy bacteria surrounded by the many possibilities that it can protect itself from metals. From the top clockwise: reduction or organic modification, blocked import of metal atoms, efflux of metal atoms, oxidation repair, metallophores binding up metal atoms, Sequestration by metal binding protein. Blue ball represents the metal atom. Not to scale

The enthusiasm around the use of metalloantimicrobials, is that bacteria were not expected to be resistant nor develop tolerance and thus would not have the same problem as organic-based antibiotics. This is somewhat ridiculous considering that bacteria would have been exposed to high metal ion concentrations during various geological events in the earth’s history as well as more recent anthropogenic exposures which led bacteria to develop survivable relationships with many metals (Maret 2016; Lemire and Turner 2017; see also book of Hurst 2022). Although this may not be the case for the new to nature metal complexes where life forms are naïve to their exposure. But for metal ions it was understood that there was resistance to toxic metals at the same time that metal ion susceptibility was explored (Sterritt and Lester 1980). As described above, studies looking into the mechanism of MBAs demonstrate that as opposed to organic antibiotics that have a single biochemical target, MBAs mechanisms of action are multi-factorial. As a result, one would not expect a single gene mutation in one of the many targets to lead to high resistance. Regardless, resistance to metal ions exists through metal resistance gene (MRG) determinants specifically evolved from exploiting or modifying normal cellular enzymes or other systems (typically efflux pumps) to provide resistance to a specific metals or metalloids (Murray et al., 1999; Hobman and Crossman 2015). Figure 2 cartoons these mechanisms with the text of description follows below.

We now have a good understanding of metal resistance genes. Although these are typically encoded on bacterial plasmids, these resistance determinants are also found on other mobile genetic elements and thus are found on chromosomes as well. MRGs encode resistance systems for specific metal ions including: Ag^+^, AsO^2−^, AsO_4_^3−^, Cd^2+^, Co^2+^, CrO_4_^2−^, Cu^2+^, Hg^2+^, Ni^2+^, Pb^2+^, Sb^3+^, TeO_3_^2−^, Tl^+^, and Zn^2+^ (Silver and Phung 1996; He et al. 2023). These MRGs are now nicely catalogued on the web-based resource bacmet.biomedicine.gu.se (Pal et al. 2014). The biochemical mechanisms of these metal-resistant determinants trend into a small number of categories:

1. An efflux pump that extrudes the metal ion outside the cell to maintain a low concentration of below toxic levels.
2. Bioconversion of the metal speciation state through either oxidation, reduction, or modification with an organic molecule to a less toxic form.
3. Sequestration by a metal-binding protein or small molecule ligands (metallophores).
4. Combination of any of the above.

Beyond these encoded metal-specific MRG resistance mechanisms, a number of ‘omic system biology studies in the past 5 years have shown that adapted tolerance occurs for both chronic and acute antimicrobial metal ion challenges as seen for studies on silver (McQuillan and Shaw 2014; Boenigk et al 2014; Saulou-Berion et al. 2015; Gugala et al 2018; Wang et al. 2019; Betts et al. 2021), Gallium (Gugala et al. 2019a, b), copper (Gugala et al. 2022). What is remarkable in these studies, is one observes that there are many different functional systems (KEGG categories) that are involved in responding to the metalloantimicrobial challenge. The **good** news within these studies is that they clearly illustrate that metals have pleiotropic effects on a bacterium cells’ physiology and that there are remarkably few shared genes in the response between different metals (Gugala et al. 2022). However, these studies also demonstrate a broad physiological response to elicit tolerance to the metal challenge which implies it will be difficult to find complementary antimicrobials to bypass tolerance or enhance the metalloantimicrobial efficacy.

Beyond the adaptive physiological response leading to tolerance indicated above, we also see biofilm related mechanisms of tolerance to metal based antimicrobials (Harrison et al. 2007). As indicated above, we learned in the good that metal ions are able to kill off persister cells in a biofilm and potentially eradicate all cells of a biofilm infection. However, the metals studied were unable to deal with phase variants/colony variants produced via the GacSA system and di-cyclic-GMP signalling in Gram-negative bacteria (Davies et al. 2007; and reviewed in Sindeldecker and Stoodley 2021) and other similar mechanisms in Gram-positives such as *Staphylococcus aureus* (reviewed in Kahl 2014). Even more concerning is not only are colony variants tolerant to metal ion challenge, but that exposure to many metal ions at sub-lethal concentrations increases the frequency of tolerant variants in the population (Workentine et al. 2010). Therefore, the choice of what metal to treat a biofilm is critical as it could lead to a greater problem of heightened antimicrobial resistance to both antibiotics and metalloantimicrobials. Additionally, the biofilm matrix, depending on its composition may act to sequester metals away or even catalyze metal speciation transformations.

As with the evolution of antibiotic resistance, the same processes will lead to the development of resistance to antimicrobial metals. Exposing bacteria to sublethal concentrations of an antimicrobial gives selective pressure towards the more tolerant cells whose physiological fitness is then selected for. Thus, any metal tolerant beneficial mutations are then carried over to daughter cells where continuing selective pressure gives rise to a cycle of a new metal resistant determinant carried in the population. Therefore, just like the misuse of antibiotics, the misuse of metalloantimicrobials will lead to the evolution of resistance (Chopra 2007). Unfortunately, there is a plethora of **bad uses** of metalloantimicrobials already on the market, and although on the surface they sound like a good idea and perhaps work for what they were marketed for, their use provides an environment for resistance to evolve.

Examples of these currently available products that are unnecessary uses and thus a **bad use** of metalloantimicrobials include but are not limited to: toothbrush bristles, deodorants, prophylactic use, probiotics, dietary supplements (gummies), agriculture pest control, plant pathogen control, toilets, biofouling control, non-medical wearable devices (smart watches), antimicrobial glass, water taps and water aerator grids, medical imaging contrast agents, facial serums, coatings or additives to non-metallic costume jewelry, cosmetic preservatives; various personal hygiene products, non-hospital use face masks. Of course, any of the **good** uses performed irresponsibly or overly scaled up will lead to issues, for example non-specifically spraying antimicrobial copper formulation on an entire crop or orchard.

If we consider these inappropriate uses of netalloantimicrobials, they may provide an explanation for the resistance that is being seen now in commercial anti-microbial silver-impregnated wound dressings used in healthcare settings. Such wound dressings were validated in the late 2000s and did not show any organisms displaying resistance; at least not for acute exposures (Lemire et al. 2017). Although silver resistance existed earlier (Percival et al. 2005), it is now common to remove a dressing that is covered with silver-resistant bacteria leading to clinical isolates tolerant to not only silver but other metals (Gugala et al. 2019a, b; Pormohammad et al. 2022). Although cause and effect are difficult to prove, one can imagine a patient who had been wearing silver-impregnated fabrics would have acquired organisms within their skin microbiome tolerant to silver.

One thing that we should consider in using metalloantimicrobias is the speciation of the metal ion related to its toxicity and bioavailability. The metallophores mentioned above generate an ligated speciation that modulates its bioavailability and the activity of the metal ion. What is often overlooked is the complexity of bacterial biochemistry can lead to the presence of thousands of small biomolecules with the ability to chelate or act as ligands to the free metal ions and change their toxicity. Additionally, the physiology of the cell and the variety of redox enzymes present can change the redox state of the metal ions that again leads to significant differences in their toxicity. These processes at this time are quite unpredictable, particularly comparing conditions in a laboratory vs. real-life infection.

Although exciting advancements are evolving in the field of metallic complexes and catalytic metallic drugs, an unfortunate aspect of these, is that many of the metal centers such as ruthenium, platinum, and iridium are rare and/or expensive and the chemistry of the organic ligands can be difficult to scale. Thus, such metalloantimicrobials in this class would likely only be useful for specialized targeted use.

## The ugly

Addressing sources of environmental pollution particularly from the pharmaceutical, agricultural and healthcare sectors is critical goals of reducing conditions for the evolution of resistance to antimicrobials. Pollution from **poor** practices of sewage and municipal waste needs to be curtailed to control resistance to typical organic antibiotics and antiseptics. **Misuse** now presents a similar problem for metalloantimicrobials. The 2023 report by the United Nations Environment Program (UNEP) recognizes that the health of people, wildlife, agriculture, and the environment are closely intertwined, what we now refer to as OneHealth. Thus, misuse in one area can have severe consequences in another.

Thus, we must consider the full system cycle of the use of metalloantimicrobials, from the mining of the metals, how green the chemical synthesis of the organic ligands are, transportation and packaging pollution, use effects leading to changes in microbiomes, and end-use effects on water treatment plants, soil, aquatic and marine systems. All these factors magnify as one moves to scale up metalloantimicrobials towards routine use in crop, animal and human health. If we consider antibiotic use in humans worldwide was ~ 14 daily doses/1000, or ~ 40 billion total daily doses in 2018 (Browne et al. 2021), then if we directly replaced with metalloantimicrobials, this would lead to a need of producing ~ 1–5 billion kgs of these metal based antimicrobials per year just for human use. Therefore, beyond just developing novel compounds and formulations, one must consider the use of stewardship and tight regulations in all jurisdictions! But it leaves one worried, like many developments, that the genie has already left the bottle.

Let’s consider in this context, as an example, the ‘**ugly**’ of Metal nanoparticles. Of the metal delivery mechanisms, delivery in a nanoparticle form has become increasingly popular. The field of nanotechnology has seen a plethora of various engineered nanomaterials (e-NM) developed for a wide variety of applications. Such e-NM can be in two categories, nano-enabled products and nano-enhanced materials, and we see antimicrobials in both categories. Engineered-NM in medicine poses some significant advantages for drug delivery as they easily enter the body through nasal/olfactory, respiratory, gastrointestinal, placenta, and brain − blood barriers, translocating in the bloodstream. On the one hand, this is ideal for antimicrobials and other drugs. On the other hand, we find MBA-NPs and other MBAs used in a remarkable number of products that are ridiculous uses of precious antimicrobials. Examples of **ugly uses** include but are not limited to: as odour control in sportswear, socks, sleepwear, bedding and other non-medically essential textiles, household furniture coatings, cosmetics, sporting goods gear (hockey, soccer, baseball, scuba diving, etc.), skin creams and lotions, household paint, laundry balls, baby clothes, shaving lotion, cat litter or litter boxes, etc.

We have a poor appreciation of the chronic consequences of the use of these products (Salieri et al. 2018). There seems to be no specific internationally agreed regulations, protocols, or legal definitions for the production, handling, labelling, toxicity testing and environmental impact assessment of NM (Jeevanandam et al. 2018). As a consequence, we have seen the unbridled explosion of use and NM on the market. For example, in 2005, there was only approximately 50 on the market and by 2020 there were more than 5000 (Hansen et al. 2020). A scary recent study identified that engineered e-NM are reaching the glymphatic and central nervous systems leading to accumulation in the brain and may be a contributor to dementia (Calderón-Garcidueñas and Ayala 2022). Additionally, we are just now starting to appreciate possible synergies, antagonisms and additivity of nanomaterials as mixtures (Zhang et al 2022).

Further, a developing problem is what happens to the e-NM post-primary use, particularly the metal-based NPs. The metalloantimicrobial NPs are typically fibre surface-bound or suspended in liquids, creams, or emulsions. Thus, such NPs can be easily released into the environment during product use and/or disposal. We are now seeing these NMs in wastewater treatment plants (WWTP) (Moeta et al. 2019). This means that the NM enters aquatic or marine systems resulting in downstream exposure and accumulation in such ecosystems. Thus we see that the WWTP acts as a gateway to release NM from products into the environment. Although little monitoring has been done to date, some observations show that 70–90% of the NM interacts and settles with the biomass sludge of the WWTP (Lazareva and Keller 2014). The remainder stays with the effluent stream directly released to the environment. In many cases worldwide, biomass sludge, if not buried in a landfill, is sterilized and used as a soil fertilizer or amendment in agriculture and through this vehicle, the metalloantimicrobial-NPs are released into the environment. Such practices could change the microbiome of agricultural soil and plants, aquatic and marine ecosystems. The NPs’ coating is key for the stability (Piacenza et al. 2018) and the interactions (Surrette et al. 2019) with biological material that governs its fate in and through the environment. Regardless, at some point, the metalloantimicrobia NPs will decompose, releasing the metal ions of its core leading to the localized pulse of metals, which is key to their anti-microbial activity, yet a concern regarding their environmental impact contributing to toxic metal pollution. Finally, a recent review suggests that there is no statistically significant advantage in wound healing improvement through the use of NMs (Pormohammad et al. 2021).

For this reason, we group NPs and particularly their over-marketing and unnecessary uses in the **ugly** category. There is **poor** to no regulations around the use of so-called ‘natural compounds’ in most of the world. Most jurisdictions consider metals in this category, particularly essential metal elements. The result is that it is relatively easy to patent applications (Sim et al. 2018) and bring a metal-based antimicrobial application to market, particularly for topical use. For example, colloidal silver (and other metalloantimicrobial NPs like TiO_2_, ZnO, etc.) are sold and marketed in most natural health food stores world-wide. Add to this the social media and ‘fake news’ eras we have evolved to and one gets ridiculous uses such as the case of Paul Karason who consumed so much nano silver that argyria particles precipitated throughout his body turning him blue.

Another issue that is not limited to metalloantimicrobials, but all antimicrobials is they are tested and validated against a single species of bacteria at a time. With most products being approved if they can demonstrate a 99.99% reduction in colony forming units, which is 3 log_10_ decrease, that is not that remarkable if one has an infection that is in the 10^7^ to 10^9^ bacteria present. But beyond this number game, it is now being increasingly observed that mixed species and microbial communities behave differently as a whole compared to individually. An example is a ~ 1000-fold increase in tolerance to silver when *Staphylococcus aureus* is co-cultured with *Pseudomonas aeruginosa* (Lemire et al. 2017; Monych and Turner 2020).

Another issue of considerable **concern** is that it has been observed for more than 10 years that metal contamination can function as a fitness pressure that contributes to the selection for antibiotic resistance (issue reviewed by: Baker-Austen et al. 2006; Vats et al. 2022). These reviews point to multiple studies that have also observed the co-occurrence of antibiotic resistance genes with metal resistance genes particularly in agricultural settings.

## Summary and some promise

The goal of this review was not to present an extensive discussion of all works in the field of metals as antimicrobials but to briefly point out the stage we’re at and the challenges we have to deal with. Metal and metalloid based compounds have remarkable antimicrobial properties and certainly have exciting potential to help in the response to multidrug resistant pathogens. Beyond this, they are also investigated for use to treat cancer multidrug resistance (Valente et al. 2021). So metals are certainly super drugs. We just need to be responsible and understand the full cycle of use with OneHealth thinking and appreciate that treating an infection in a human could affect aquatic environments and exposures by agricultural practices.

Regardless, metal-based drug development is still very much in its earlier stages. There is tremendous potential for organic compounds combined with metals. This is possible due to decades of research in organo-metallic chemistry. From this, likely metalloantimicrobials are our best hope to avoid the AMR cliff if one can apply our wealth of chemical knowledge to select cheaper and more readily available metal elements with target functionalized organic ligands. As we saw above, arsenic has been used historically in traditional medicines. Many groups now propose revisiting organo-arsenicals with proven efficacy to combat emerging pathogens (Paul et al. 2023). Creative chemical design should lead to effective and safe arsenical drugs.

The hidden problem in the past was using a single antimicrobial acting on a single target. This allows for mutations in this single target to provide resistance. This of course worked for many years, but it also allowed for easy evolution given only the mutations in that single target are necessary for resistance. Thus, it is crucial that we use mixtures of antimicrobials that target the cell in different ways to avoid easily evolved resistance. As such, the solution for our AMR era could be as simple as combining novel approaches as well as our ancient knowledge. We have seen that many metals can act synergistically (Pormohammad et al. 2020) and that we do not see resistance evolving to the dual metal exposure (Pormohammad et al. 2022, 2023). Such synergistic mixtures leads to efficacy at much lower concentrations and thus, lower environmental contamination. One could imagine mixtures of metalloantimicrobials and their mixtures formulating with other organic based antimicrobials leading to the resulting antimicrobial concoction hitting the microbe in a multitude of ways.

## References

[CR1] Abbaszadegan A, Ghahramani Y, Gholami A, Hemmateenejad B, Dorostkar S, Nabavizadeh M, Shargh H (2015). The effect of charge at the surface of silver nanoparticles on antimicrobial activity against gram-positive and gram-negative bacteria: a preliminary study. J Nanomater.

[CR2] Alexander JW (2009). History of the medical use of silver. Surg Infect.

[CR3] Baker-Austin C, Wright MS, Stepanauskas R, McArthur JV (2006). Co-selection of antibiotic and metal resistance. Trends Microbiol.

[CR4] Balaure PC, Grumezescu AM (2020). Recent advances in surface nanoengineering for biofilm prevention and control. Part II: active, combined active and passive, and smart bacteria-responsive antibiofilm nanocoatings. Nanomaterials (basel).

[CR5] Betts HD, Neville SL, McDevitt CA, Sumby CJ, Harris HH (2021). The biochemical fate of Ag^+^ ions in Staphylococcus aureus, Escherichia coli, and biological media. J Inorg Biochem.

[CR6] Bhardwaj AK, Vinothkumar K, Rajpara N (2013). Bacterial quorum sensing inhibitors: attractive alternatives for control of infectious pathogens showing multiple drug resistance. Recent Pat Antiinfect Drug Discov.

[CR7] Bijelic A, Aureliano M, Rompel A (2018). The antibacterial activity of polyoxometalates: structures, antibiotic effects and future perspectives. Chem Commun (Camb)..

[CR8] Boenigk J, Beisser D, Zimmermann S, Bock C, Jakobi J, Grabner D, Groβmann L, Rahmann S, Barcikowski S, Sures B (2014). Effects of silver nitrate and silver nanoparticles on a planktonic community: general trends after short-term exposure. PLoS ONE.

[CR9] Borkow G, Gabbay J (2009). Copper, an ancient remedy returning to fight microbial, fungal and viral infections. Curr Chem Biol.

[CR10] Boyd NK, Teng C, Frei CR (2021). Brief overview of approaches and challenges in new antibiotic development: a focus on drug repurposing. Front Cell Infect Microbiol.

[CR11] Braga D (2023). Crystal engineering: from promise to delivery. Chem Commun.

[CR12] Braga D, Casali L, Grepioni F (2022). The relevance of crystal forms in the pharmaceutical field: sword of damocles or innovation tools?. Int J Mol Sci.

[CR13] Brown CL, Smith K, McCaughey L, Walker D (2012). Colicin-like bacteriocins as novel therapeutic agents for the treatment of chronic biofilm-mediated infection. Biochem Soc Trans.

[CR14] Browne AJ, Chipeta MG, Haines-Woodhouse G, Kumaran EPA, Hamadani BHK, Zaraa S, Henry NJ, Deshpande A, Reiner RC, Day NPJ, Lopez AD, Dunachie S, Moore CE, Stergachis A, Hay SI, Dolecek C (2021). Global antibiotic consumption and usage in humans, 2000–18: a spatial modelling study. Lancet Planet Health.

[CR15] Calderón-Garcidueñas L, Ayala A (2022). Air pollution, ultrafine particles, and your brain: are combustion nanoparticle emissions and engineered nanoparticles causing preventable fatal neurodegenerative diseases and common neuropsychiatric outcomes?. Environ Sci Technol.

[CR16] Chandrangsu P, Rensing C, Helmann J (2017). Metal homeostasis and resistance in bacteria. Nat Rev Microbiol.

[CR17] Chang T, Babu RP, Zhao W, Johnson CM, Hedström P, Odnevall I, Leygraf C (2021). High-resolution microscopical studies of contact killing mechanisms on copper-based surfaces. ACS Appl Mater Interfaces.

[CR18] Chopra I (2007). The increasing use of silver-based products as antimicrobial agents: a useful development or a cause for concern?. J Antimicrob Chemother.

[CR19] Cieplik F, Deng D, Crielaard W, Buchalla W, Hellwig E, Al-Ahmad A, Maisch T (2018). Antimicrobial photodynamic therapy—what we know and what we don’t. Crit Rev Microbiol.

[CR20] Dadgostar P (2019). Antimicrobial resistance: implications and costs. Infect Drug Resist.

[CR21] Davies D (2003). Understanding biofilm resistance to antibacterial agents. Nat Rev Drug Discov.

[CR22] Davies JA, Harrison JJ, Marques LR, Foglia GR, Stremick CA, Storey DG, Turner RJ, Olson ME, Ceri H (2007). The GacS sensor kinase controls phenotypic reversion of small colony variants isolated from biofilms of Pseudomonas aeruginosa PA14. FEMS Microbiol Ecol.

[CR23] De Morgan C (1870). On the treatment of gun-shot wounds by chloride of zinc. Br Med J.

[CR24] Duffus JH (2002). “Heavy metals”—a meaningless term?. Pure Appl Chem.

[CR25] Francolini I, Donelli G (2010). Prevention and control of biofilm-based medical-device-related infections. FEMS Immunol Med Microbiol.

[CR26] Frankel ML, Demeter MA, Lemire J, Turner RJ (2016). Evaluating metal tolerance capacity of microbial communities isolated from the Alberta oil sands processed water. PLoS ONE.

[CR27] Frei A (2020). Metal complexes, an untapped source of antibiotic potential?. Antibiotics (basel).

[CR28] Frei A, Zuegg J, Elliott AG, Baker M, Braese S, Brown C, Chen F, Dowsonc G, Dujardin G, Jung N, King AP, Mansour AM, Massi M, Moat J, Mohamed HA, Renfrew AK, Rutledge PJ, Sadler PJ, Todd MH, Willans CE, Wilson JJ, Cooper MA, Blaskovich MAT (2020). Metal complexes as a promising source for new antibiotics. Chem Sci.

[CR29] Frei A, Verderosa AD, Elliott AG, Zuegg J, Blaskovich MAT (2023). Metals to combat antimicrobial resistance. Nat Rev Chem.

[CR30] Garcia Jimenez D, Poongavanam V, Kihlberg J (2023). Macrocycles in drug discovery─learning from the past for the future. J Med Chem.

[CR31] Gugala N, Turner RJ, Rai M, Ingle A, Medici S (2018). The potential of metals in combating bacterial pathogens. Biomedical applications of metals.

[CR32] Gugala N, Lemire JJ, Turner RJ (2017). The efficacy of different anti-microbial metals at preventing the formation of, and eradicating bacterial biofilms of pathogenic indicator strains. J Antibiot.

[CR33] Gugala N, Lemire JA, Chatfield-Reed K, Yan Y, Chua G, Turner RJ (2018). Using a chemical genetic screen to enhance our understanding of the antibacterial properties of silver. Genes.

[CR34] Gugala N, Chatfield-Reed K, Turner RJ, Chua G (2019). Using a chemical genetic screen to enhance our understanding of the antimicrobial properties of gallium against Escherichia coli. Genes.

[CR35] Gugala N, Vu D, Parkins MD, Turner RJ (2019). Specificity in the susceptibilities of Escherichia coli, Pseudomonas aeruginosa, and Staphylococcus aureus clinical isolates to six metal antimicrobials. Antibiotics.

[CR36] Gugala N, Salazar-Alemán DA, Chua G, Turner RJ (2022). Using a chemical genetic screen to enhance our understanding of the antimicrobial properties of copper. Metallomics.

[CR37] Hamadani BH, KumaranEAP MB, Achalapong S, Agarwal R (2022). Global burden of bacterial antimicrobial resistance in 2019: a systematic analysis. Lancet.

[CR38] Hansen SF, Hansen OFH, Nielsen MB (2020). Advances and challenges towards consumerization of nanomaterials. Nat Nanotechnol.

[CR39] Harrison JJ, Ceri H, Stremick CA, Turner RJ (2004). Biofilm susceptibility to metal toxicity. Environ Microbiol.

[CR40] Harrison JJ, Turner RJ, Ceri H (2005). Persister cells, the biofilm matrix, and tolerance to metal cations in biofilm and planktonic pseudomonas aeruginosa. Environ Microbiol.

[CR41] Harrison JJ, Ceri H, Turner RJ (2007). Multimetal resistance and tolerance in microbial biofilms. Nat Rev Microbiol.

[CR42] Harrison JJ, Tremaroli V, Stan MA, Chan CS, Vacchi-Suzzi C, Heyne BJ, Parsek MR, Ceri H, Turner RJ (2009). Chromosomal antioxidant genes have metal ion-specific roles as determinants of bacterial metal tolerance. Environ Microbiol.

[CR43] He Z, Shen J, Li Q, Yang Y, Zhang D, Pan X (2023). Bacterial metal(loid) resistance genes (MRGs) and their variation and application in environment: a review. Sci Total Environ.

[CR44] Hobman JL, Crossman LC (2015). Bacterial antimicrobial metal ion resistance. J Med Microbiol.

[CR45] Hodges NDC (1889). The value of mercuric chloride as a disinfectant. Science.

[CR46] Hurst CH (2022). Microbial metabolism of metals and metalloids. Advances in environmental microbiology, volume 10.

[CR47] Jeevanandam J, Barhoum A, Chan YS, Dufresne A, Danquah MK (2018). Review on nanoparticles and nanostructured materials: history, sources, toxicity and regulations. Beilstein J Nanotechnol.

[CR48] Jonathan S, McQuillan JS, Shaw AM (2014). Differential gene regulation in the Ag nanoparticle and Ag+-induced silver stress response in Escherichia coli: A full transcriptomic profile. Nanotoxicology.

[CR49] Josefsen LB, Boyle RW (2008). Photodynamic therapy and the development of metal-based photosensitisers. Met Based Drugs.

[CR50] Kahl BC (2014). Small colony variants (SCVs) of Staphylococcus aureus–a bacterial survival strategy. Infect Genet Evol.

[CR51] Kenney GE, Rosenzweig AC (2018). Chalkophores. Annu Rev Biochem.

[CR52] Kessi J, Turner RJ, Zannoni D (2022). Tellurite and Selenite: how can these two oxyanions be chemically different yet so similar when they are processed by bacteria?. Biol Res (chile).

[CR53] Khan MR, Fromm KM, Rizvi TF, Giese B, Ahmad F, Turner RJ, Füeg M, Marsili E (2020). Metal nanoparticle-microbe interactions: synthesis and antimicrobial effects. Part & Part Syst Charact.

[CR54] Kraemer SM, Duckworth OW, Harrington JM, Schenkeveld WD (2015). Metallophores and trace metal biogeochemistry. Aquat Geochem.

[CR55] Lai S, Zhang Q, Jin L (2022). Natural and man-made cyclic peptide-based antibiotics. Antibiotics (basel).

[CR56] Laws M, Shaaban A, Rahman KM (2019). Antibiotic resistance breakers: current approaches and future directions. FEMS Microbiol Rev.

[CR57] Lazareva A, Keller AA (2014). Estimating potential life cycle releases of engineered nanomaterials from wastewater treatment plants. ACS Sustain Chem Eng.

[CR58] Lekhan A, Fiore C, Shemchuk O, Grepioni F, Braga D, Turner RJ (2022). Comparison of antimicrobial and antibiofilm activity of proflavine co-crystallized with silver, copper, zinc, and gallium salts. ACS Appl Bio Mater.

[CR59] Lemire J, Turner RJ, Das S, Dash JR (2017). Biochemical pathways in bacteria to survive metal contaminated environments. handbook of metal-microbe interactions and bioremediation.

[CR60] Lemire J, Harrison JJ, Turner RJ (2013). Antimicrobial activity of metals: mechanisms, molecular targets and applications. Nat Rev Microbiol.

[CR61] Lemire JJ, Kalan L, Gugala N, Bradu A, Turner RJ (2017). Silver oxynitrate—an efficacious compound for the prevention and eradication of dual –species biofilms. Biofouling.

[CR62] Leslie M, Fadaak R, Lethebe BC, Szostakiwskyj JH (2023). Assessing the appropriateness of community-based antibiotic prescribing in Alberta, Canada, 2017–2020, using ICD-9-CM codes: a cross-sectional study. CMAJ Open.

[CR63] Lewis K (2010). Persister cells. Annu Rev Microbiol.

[CR64] Li F, Collins J, Keene FR (2015). Ruthenium complexes as antimicrobial agents. Chem Soc Rev.

[CR65] Li R, Chen T, Pan X (2021). Metal-organic-framework-based materials for antimicrobial applications. ACS Nano.

[CR66] Li YP, Ben Fekih I, Chi Fru E, Moraleda-Munoz A, Li X, Rosen BP, Yoshinaga M, Rensing C (2021). Antimicrobial activity of metals and metalloids. Annu Rev Microbiol.

[CR67] Liew KB, Janakiraman AK, Sundarapandian R, Khalid SH, Razzaq FA, Ming LC, Khan A, Kalusalingam A, Ng PW (2022). A review and revisit of nanoparticles for antimicrobial drug delivery. J Med Life.

[CR68] Magana M, Pushpanathan M, Santos AL, Leanse L, Fernandez M, Ioannidis A, Giulianotti MA, Apidianakis Y, Bradfute S, Ferguson AL, Cherkasov A, Seleem MN, Pinilla C, de la Fuente-Nunez C, Lazaridis T, Dai T, Houghten RA, Hancock REW, Tegos GP (2020). The value of antimicrobial peptides in the age of resistance. Lancet Infect Dis.

[CR69] Maret W (2016). The metals in the biological periodic system of the elements: concepts and conjectures. Int J Mol Sci.

[CR70] Mercan D-A, Niculescu A-G, Grumezescu A (2022). Nanoparticles for antimicrobial agents delivery—an up-to-date review. Int J Mol Sci.

[CR71] Moeta PJ, Wesley-Smith J, Maity A, Thwala M (2019). Nano-enabled products in South Africa and the assessment of environmental exposure potential for engineered nanomaterials. SN Appl Sci..

[CR72] Mohanta YK, Chakrabartty I, Mishra AK, Chopra H, Mahanta S, Avula SK, Patowary K, Ahmed R, Mishra B, Mohanta TK, Saravanan M, Sharma N (2023). Nanotechnology in combating biofilm: a smart and promising therapeutic strategy. Front Microbiol.

[CR73] Monych NK, Turner RJ (2020). Multiple compounds secreted by Pseudomonas aeruginosa increase the tolerance of Staphylococcus aureus to the antimicrobial metals copper and silver. Systems.

[CR74] Monych N, Gugala N, Turner RJ, Domb AJ, Kunduru KR, Shady F (2019). Metal-based antimicrobials. Biomaterials science series No. 5. Antimicrobial materials for biomedical applications.

[CR75] Mubeen B, Ansar AN, Rasool R, Ullah I, Imam SS, Alshehri S, Ghoneim MM, Alzarea SI, Nadeem MS, Kazmi I (2021). Nanotechnology as a novel approach in combating microbes providing an alternative to antibiotics. Antibiotics (basel).

[CR76] Murray CJL, Ikuta KS, Sharara F, Swetschinski L, Robles Aguilar G, Gray A, Han C, Bisignano C, Rao P, Wool E, Johnson SC, Browne AJ, Chipeta MG, Fell F, Hackett S, Haines-Woodhouse G, Kashef NDH (1999). Microbial, heavy-metal resistance. Appl Microbial Biotechnol.

[CR77] OneCDC (2020) https://blogs.cdc.gov/global/2020/11/02/one-health-a-comprehensive-approach-to-preventing-disease-saving-lives/. Accessed 10 Aug 2023

[CR78] Pal C, Bengtsson-Palme J, Rensing C, Kristiansson E, Larsson DG (2014). BacMet: antibacterial biocide and metal resistance genes database. Nucleic Acids Res.

[CR79] Pandey A, Śmiłowicz D, Boros E (2021). Galbofloxacin: a xenometal-antibiotic with potent *in vitro* and *in vivo* efficacy against *S. aureus*. Chem Sci.

[CR80] Patra M, Gasser G, Metzler-Nolte N (2012). Small organometallic compounds as antibacterial agents. Dalton Trans.

[CR81] Paul NP, Galván AE, Yoshinaga-Sakurai K, Rosen BP, Yoshinaga M (2023). Arsenic in medicine: past, present and future. Biometals.

[CR82] Percival SL, Bowler P, Russell D (2005). Bacterial resistance to silver in *wound care*. J Hosp Infect.

[CR83] Pereira J (1836). Materia medica or pharmacology and general therapeutics. Lond Med Gaz.

[CR84] Piacenza E, Presentato A, Turner RJ (2018). Stability of biogenic metal(loid) nanomaterial related to the colloidal stabilization theory of chemical nanostructures. Crit Rev Biotechnol.

[CR85] Plotniece A, Sobolev A, Supuran CT, Carta F, Björkling F, Franzyk H, Yli-Kauhaluoma J, Augustyns K, Cos P, De Vooght L, Govaerts M, Aizawa J, Tammela P, Žalubovskis R (2023). Selected strategies to fight pathogenic bacteria. J Enzyme Inhib Med Chem.

[CR86] Pormohammad A, Turner RJ (2020). Silver antibacterial synergism activities with eight other metal(loid)-based antimicrobials against escherichia coli, pseudomonas aeruginosa, and staphylococcus aureus. Antibiotics.

[CR87] Pormohammad A, Monych NK, Ghosh S, Turner DL, Turner RJ (2021). Nanomaterials in wound healing and infection control. Antibiotics.

[CR88] Pormohammad A, Greening D, Turner RJ (2022). Synergism inhibition and eradication activity of silver nitrate-potassium tellurite combination against *Pseudomonas aeruginosa* biofilms. J Antimicrob Chemother.

[CR89] Pormohammad A, Firrincieli A, Salazar-Aleman DA, Mohammadi M, Hansen DD, Cappelletti M, Zannoni D, Turner RJ (2023). Insights into the synergistic antibacterial activity of silver nitrate with potassium tellurite against *Pseudomonas aeruginosa*. Microbiol Spectr.

[CR90] Pourret O, Hursthouse A (2019). It’s time to replace the term “Heavy Metals” with “Potentially Toxic Elements” when reporting environmental research. Int J Environ Res Public Health.

[CR91] Pourret O, Bollinger JC, Hursthouse A (2021). Heavy metal: a misused term?. Acta Geochim.

[CR92] Presentato A, Turner RJ, Vasquez CC, Yurkov V, Zannoni D (2019). Tellurite-dependent blackening of bacteria is out from the dark-age!. Environ Chem.

[CR93] Russell PE (2005). A century of fungicide evolution. J Agric Sci.

[CR94] Saha M, Sarkar S, Sarkar B, Sharma BK, Bhattacharjee S, Tribedi P (2016). Microbial siderophores and their potential applications: a review. Environ Sci Pollut Res Int.

[CR95] Salazar-Alemán DA, Turner RJ, Hurst CJ (2022). Metal based antimicrobials—uses and challenges. In microbial metabolism of metals and metalloids. Advances in environmental microbiology.

[CR96] Salgado CD, Sepkowitz KA, John JF, Cantey JR, Attaway HH, Freeman KD, Sharpe PA, Michels HT, Schmidt MG (2013). Copper surfaces reduce the rate of healthcare-acquired infections in the intensive care unit. Infect Control Hosp Epidemiol.

[CR97] Salieri B, Turner DA, Nowack B, Hischier R (2018). Life cycle assessment of manufactured nanomaterials: where are we?. NanoImpact.

[CR98] Saulou-Bérion C, Gonzalez S-BC, Enjalbert B, Audinot J-N, Fourquaux I, Jamme F (2015). Escherichia coli under ionic silver stress: an integrative approach to explore transcriptional, physiological and biochemical responses. PLoS ONE.

[CR99] Schwarz C, Mathieu J, Laverde Gomez JA, Yu P, Alvarez PJJ (2022). Renaissance for phage-based bacterial control. Environ Sci Technol.

[CR100] Shakoor S, Platts-Mills JA, Hasan R (2019). Antibiotic-resistant enteric infections. Infect Dis Clin North Am.

[CR101] Sharma A, Gupta VK, Pathania R (2019). Efflux pump inhibitors for bacterial pathogens: From bench to bedside. Indian J Med Res.

[CR102] Shen M, Forghani F, Kong X, Liu D, Ye X, Chen S, Ding T (2020). Antibacterial applications of metal-organic frameworks and their composites. Compr Rev Food Sci Food Saf.

[CR103] Shukla R, Peoples AJ, Ludwig KC, Maity S, Derks MGN, De Benedetti S, Krueger AM, Vermeulen BJA, Harbig T, Lavore F, Kumar R, Honorato RV, Grein F, Nieselt K, Liu Y, Bonvin AMJJ, Baldus M, Kubitscheck U, Breukink E, Achorn C, Nitti A, Schwalen CJ, Spoering AL, Ling LL, Hughes D, Lelli M, Roos WH, Lewis K, Schneider T, Weingarth M (2023). An antibiotic from an uncultured bacterium binds to an immutable target. Cell.

[CR104] Silver S, Phung LT (1996). Bacterial heavy metal resistance: new surprises. Annu Rev Microbiol.

[CR105] Sim W, Barnard RT, Blaskovich MAT, Ziora ZM (2018). Antimicrobial silver in medicinal and consumer applications: a patent review of the past decade (2007–2017). Antibiotics (basel).

[CR106] Sindeldecker D, Stoodley P (2021). The many antibiotic resistance and tolerance strategies of Pseudomonas aeruginosa. Biofilm.

[CR107] Sterritt RM, Lester JN (1980). Interactions of heavy metals with bacteria. Sci Total Environ.

[CR108] Sun Y, Zheng L, Yang Y, Qian X, Fu T, Li X, Yang Z, Yan H, Cui C, Tan W (2020). Metal-organic framework nanocarriers for drug delivery in biomedical applications. Nanomicro Lett.

[CR109] Surette MC, Nason JA, Kaegi R (2019). The influence of surface coating functionality on the aging of nanopartilces in wastewater. Environ Sci Nano.

[CR110] Teitzel GM, Parsek MR (2003). Heavy metal resistance of biofilm and planktonic Pseudomonas aeruginosa. Appl Environ Microbiol.

[CR111] Townsend T, Sololo-Gabriele H (2006). Environmental impacts of treated wood.

[CR112] Turner RJ (2017). Metal-based antimicrobial strategies. Microbial. Biotechnol.

[CR113] Valente A, Podolski-Renić A, Poetsch I, Filipović N, López Ó, Turel I, Heffeter P (2021). Metal- and metalloid-based compounds to target and reverse cancer multidrug resistance. Drug Resist Updat.

[CR114] Vats P, Kaur UJ, Rishi P (2022). Heavy metal-induced selection and proliferation of antibiotic resistance: a review. J Appl Microbiol.

[CR115] Vincent M, Duval RE, Hartemann P, Engels-Deutsch M (2018). Contact killing and antimicrobial properties of copper. J Appl Microbiol.

[CR116] Wang H, Yan A, Liu Z, Yang X, Xu Z, Wang Y (2019). Deciphering molecular mechanism of silver by integrated omic approaches enables enhancing its antimicrobial efficacy in E. coli. PLoS Biol.

[CR117] Waters JE, Stevens-Cullinane L, Siebenmann L, Hess J (2023). Recent advances in the development of metal complexes as antibacterial agents with metal-specific modes of action. Curr Opin Microbiol.

[CR118] Weng C, Tan YLK, Koh WG, Ang WH (2023). Harnessing transition metal scaffolds for targeted antibacterial therapy. Angew Chem Int Ed Engl.

[CR119] Wińska K, Mączka W, Łyczko J, Grabarczyk M, Czubaszek A, Szumny A (2019). Essential oils as antimicrobial agents-myth or real alternative?. Molecules.

[CR120] Workentine ML, Harrison JJ, Stenroos PU, Ceri H, Turner RJ (2008). Pseudomonas fluorescens view of the periodic table. Environ Microbiol.

[CR121] Workentine ML, Harrison JJ, Weljie AM, Tran VA, Stenroos PU, Tremaroli V, Vogel HJ, Ceri H, Turner RJ (2010). Phenotypic and metabolic profiling of colony morphology variants evolved from Pseudomonas fluorescens biofilms. Environ Microbiol.

[CR122] World Health Organization (2014) Antimicrobial resistance: global report on surveillance. Geneva: https://apps.who.int/iris/handle/10665/112642. Accessed 20 July 2023

[CR123] Yusuf VF, Malek NI, Kailasa SK (2022). Review on metal-organic framework classification, synthetic approaches, and influencing factors: applications in energy, drug delivery, and wastewater treatment. ACS Omega.

[CR124] Zhang F, Wang Z, Peijnenburg WJGM, Vijver MG (2022). Review and prospects on the ecotoxicity of mixtures of nanoparticles and hybrid nanomaterials. Environ Sci Technol.

